# Large Pleural Metastases With Significant Inter-fractional Volume Reduction During Online Adaptive Radiotherapy: A Case Report With Dosimetry Comparison

**DOI:** 10.7759/cureus.68407

**Published:** 2024-09-01

**Authors:** Yu-Rou Chiou, Ting Chun Lin, Jin-Huei Ji, An-Cheng Shiau, Chi-Hsien Huang, Ji-An Liang

**Affiliations:** 1 Department of Radiation Oncology, China Medical University Hospital, Taichung City, TWN; 2 Graduate Institute of Biomedical Sciences, China Medical University, Taichung City, TWN; 3 Department of Biomedical Imaging and Radiological Sciences, National Yang‐Ming University, Taipei, TWN

**Keywords:** tumor volume reduction, pleural metastases, ethos platform, dose uncertainty, inter-fractional tumor volume, treatment planning, synthetic ct, cbct, plan evaluation, online adaptive radiotherapy

## Abstract

Online adaptive radiotherapy (oART) dose calculation relies on synthetic computed tomography (sCT), which notably influences anatomical changes. This study elucidates how sCT may respond to significant inter-fractional tumor volume reduction and its subsequent impact on dose distribution. In this case report, we exported sCT and cone-beam CT (CBCT) images from each treatment session. We retrospectively analyzed 20 adaptive and scheduled plans of a patient receiving oART for large pleural metastases with notable inter-fractional tumor regression. By overriding the CT number of the dissipated tumor volume with that of the lungs on each sCT, we recalculated each plan. We compared the dose distribution between the adaptive and scheduled plans. Percentage dose difference and 3D gamma analysis were employed to assess dose variability. Results of the dose analysis showed that, compared to the online (non-overridden) plans, the recalculated plans using overridden sCT demonstrated right-shifted dose-volume histogram curves for the targets and right lung, with a slight but statistically significant increase of no less than 1.5% in *D*_mean_ and *D*_max_ for the targets and right lung. The location of hotspots shifted in alignment with tumor shrinkage and beam arrangement. Both recalculated adaptive and scheduled plans achieved ideal GTV, CTV, and PTV coverage, with adaptive plans significantly reducing the dose and irradiated volume to the right lung. In conclusion, as the pleural tumor volume decreased, online plans slightly underestimated the dose distribution and shifted the location of hotspots, though this remained clinically acceptable. Importantly, adaptive plans significantly minimized the irradiated volume of the critical OAR (right lung) while ensuring optimal dose coverage of the target volume, demonstrating the potential of sCT and adaptive oART to enhance treatment precision and efficacy in dynamically changing tumor environments.

## Introduction

Online adaptive radiotherapy (oART) allows physicians and physicists to modify patients’ contours daily and choose the optimal plan according to variation [1,2]. For cone-beam computed tomography (CBCT)-based oART, the dose calculation of the daily adaptive plans relies on synthetic computed tomography (sCT), which is generated by transforming the Hounsfield unit of simulation CT to the geometry of daily CBCT using deformable image registration [3,4]. Although oART has been applied to various anatomical sites, concerns remain about its accuracy and robustness in planning evaluations. Inferior CBCT quality can lead to distortions in the geometry of the sCT, resulting in inaccuracies in dose calculation [5,6]. Studies have reported successful implementation of oART in the abdomen and pelvic region; however, the technique is less frequently used in lung tumor cases due to the commonly encountered inconvenience in respiratory control and changes in tumor and fluid volumes leading to poor CBCT image quality [7,8].

A recently published paper provided clinical recommendations for commissioning the oART platform and simulated gas and body thickness changes on a phantom to evaluate the dose uncertainty [9]. Although changes in body size and gas can be simulated using a phantom, gross tumor volume (GTV) changes cannot be easily emulated. In this study, we analyzed the oART plans of a clinical case with extensive right-sided pleural metastasis. The patient received 40 Gy in 20 fractions to the pleural tumor, and we noticed >50% GTV reduction during the treatment course. After exporting the adaptive plans and the sCT for each plan, we overrode the shrunken tumor volume on the sCT with the lung density and then recalculated the plans to compare the daily dose difference between the overridden plans and non-overridden plans. We also plotted dose-volume histograms (DVH) for the evaluation of multiple plans.

This study thoroughly analyzed the dosimetric data of a clinical case that exhibited significant inter-fractional target volume reduction during lung oART. Through this case report, we evaluated the impact of substantial target volume changes on CBCT-based sCT and compared the dosimetry of daily adaptive plans with scheduled plans.

Part of this article was previously posted under a Creative Commons Attribution license (CC BY) on September 25, 2023.

## Case presentation

Clinical case and ethics approval

A 60-year-old gentleman with metastatic salivary gland adenoid cystic carcinoma received 40 Gy in 20 fractions to the progressive massive right pleural metastases. He had no dyspnea and could comply with the breath-hold technique, and CBCT-based oART was arranged for the 20-fraction treatment. The study was approved by the research ethics committee of our institute (CMUH106-REC3-119(CR-5)).

oART treatment planning and treatment delivery

A CT simulator (Siemens SOMATOM Definition AS 64-slice Configuration, Erlangen, Germany) and respiratory gating system (SDX™ Spirometric Motion Management System, Dyn’R, Toulouse, France) were employed to acquire images for treatment planning. The DIBH technique was applied for CT simulation and all treatment sessions. We applied the fallback planning method, in which a physicist designed a nine-field intensity-modulated radiation therapy (IMRT) plan in the Eclipse system (Siemens/Varian, version 16.01.10, Palo Alto, CA). This plan was then imported to the Ethos system (Varian, version 1.1) for dose calculation and plan optimization. The criteria for dose coverage are listed in Table 1. At least 99% of GTV should be covered by 99% of the prescribed dose. At least 90% of the clinical target volume (CTV) and planning target volume (PTV) were covered by 95% of the prescribed dose. The volume should be less than 0.03 cm3 for over 105% of the prescribed dose. For organs at risk (OARs), the mean heart dose should be less than 950 cGy.

**Table 1:** Constraint table for the treatment planning on the Ethos platform. GTV: gross tumor volume; CTV: clinical target volume; PTV: planning target volume. *V_99%_ > 99% means that 99% of the target volume should be covered by 99% of the prescribed dose. **D_0.03cm^3^_ < 105% means that the dose of 0.03 cm^3^ target volume should be less than 105% of the prescribed dose. ***D_mean_ is the mean dose of the ROI (region of interest).

Structure	Ethos clinical goals	Variation	Ethos priority
GTV	*V*_99% _> 99% ^*^	$\geq$ 98%	1
CTV	*V*_90%_ > 95%		1
	*D*_0.03cm^3^_ < 105% ^**^	$\leq$ 107%	1
PTV	*V*_90%_ > 95%		1
	*D*_0.03cm^3^_ < 105%	$\leq$ 107%	1
Heart	*D*_mean_ < 950 cGy ^***^		2
	*D*_0.03cm^3^_ < 3100 cGy		2
Lungs	*V*_2000cGy_ $\leq$ 23%		2
	*D*_mean_ $\leq$ 1000 cGy		2
Spinal cord	*D*_0.03cm__^3^_ < 3000 cGy		2

At the beginning of each treatment session, a breath-hold 3D CBCT scan was done, and influencer structures were generated. Usually, organs with a strong influence on target structures in terms of position, shape, and dosimetric impact are designated as influencer structures. For this clinical case, the influencer structures were the right lung, left lung, and heart. A physician confirmed and modified the contours of the influencers and targets as needed. The Ethos system then optimized and calculated the dose of the scheduled and adaptive plans. The physician chose the preferred plan, and the physicist performed an online plan quality assurance with Mobius 3D. After the plan was checked, a treatment session was performed.

Post-treatment plan evaluation and comparison

**Figure 1:**
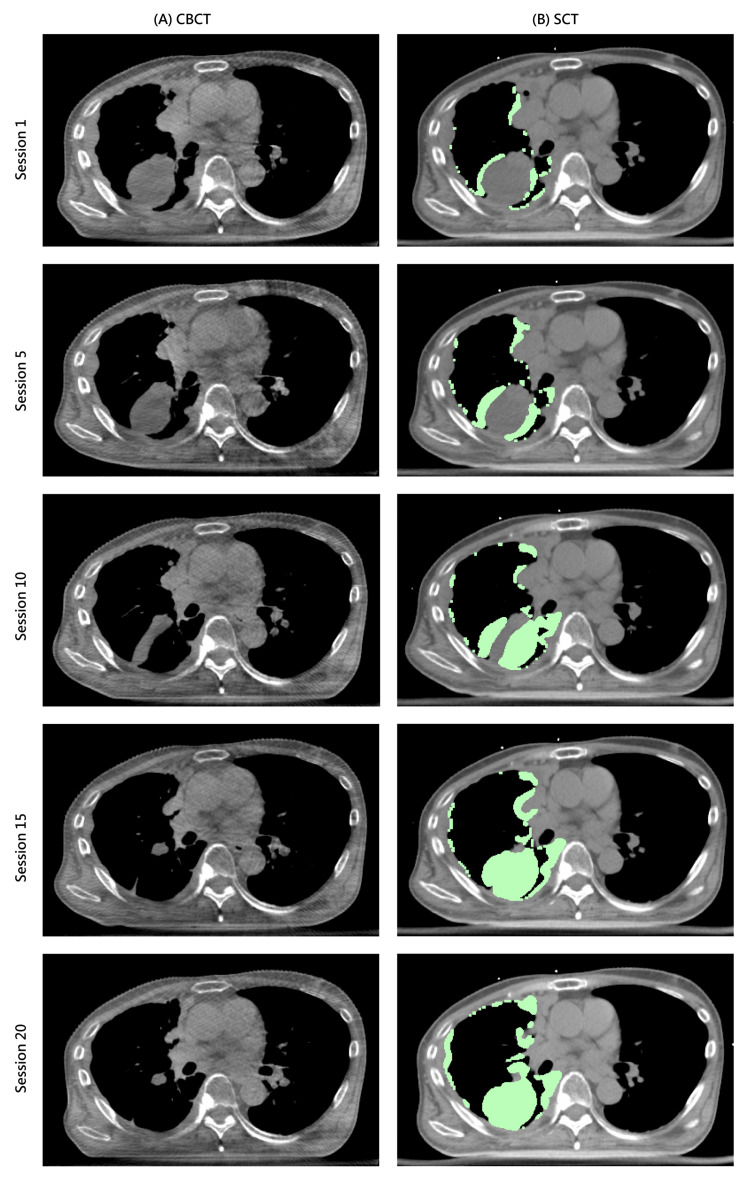
The (A) CBCT and (B) sCT of the 1st, 5th, 10th, 15th and 20th RT session. The overridden area is shown in light green. CBCT: cone-beam computed tomography; sCT: synthetic computed tomography.

After each RT session, the sCT images from each treatment session were reviewed. As sCT cannot be visualized on the couch, we retrospectively found that it could not fully adapt to the anatomical changes in the rapidly shrinking tumor. To evaluate the possible dose error caused by discrepancies in CT images, we exported CBCT and sCT from the Ethos platform to Eclipse TPS. To ensure a more accurate calculation of dose distribution that reflects the volume reduction not captured by sCT, we re-contoured the lung volumes on each session’s CBCT and sCT images according to the Hounsfield unit of the lung tissue. The lung volume difference between CBCT and sCT depicted a reduced tumor volume (Figure 1). On each session’s sCT, we overrode the reduced tumor volume with the density of the lung and then recalculated the dose for both scheduled and adaptive plans on the overridden sCT. We plotted the DVH curves for the targets and the right lung of the 80 treatment plans (20 adaptive plans and 20 scheduled plans, both original and recalculated). Additionally, we compared the *D*_max_ and *D*_mean_ of the original and recalculated adaptive and scheduled plans using a paired t-test. The dose distributions of the original and recalculated plans were compared using 3D gamma index analysis with gamma criteria of 1 mm/3%, 2 mm/3%, 3 mm/3%, and 2 mm/2%. Gamma index analysis was determined using the Dose Comparison module of 3D Slicer (version 5.6.2).

GTV reduction and sCT geometry

**Figure 2:**
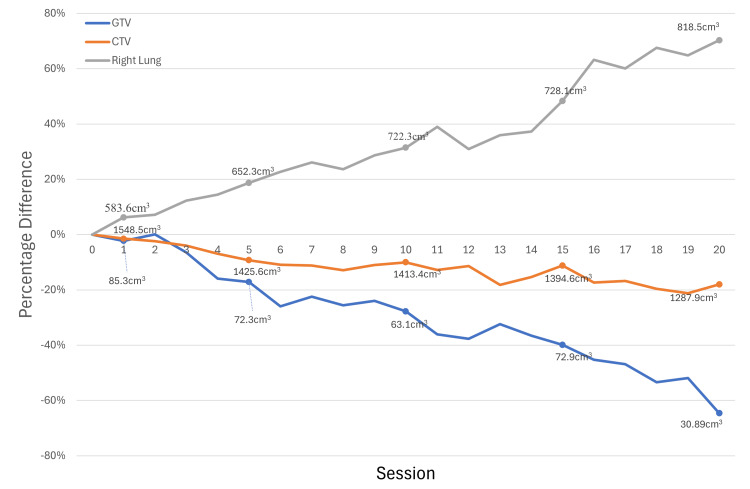
Inter-fractional change of the GTV, CTV, and right lung volume over the 20 fractions. GTV: gross tumor volume; CTV: clinical target volume.

**Figure 3:**
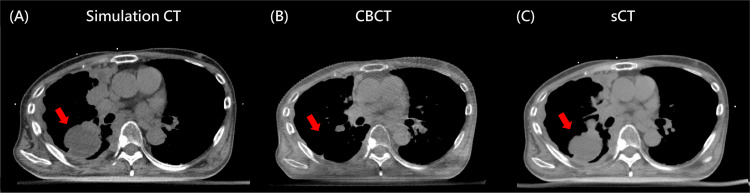
Representative images from the 20th treatment session for the (A) simulation CT, (B) on-couch CBCT, and (C) synthetic CT. CBCT: cone-beam computed tomography; sCT: synthetic computed tomography.

During the 5th fraction, a GTV reduction of 18% was observed. In the 10th, 15th, and 20th fractions, the rates of GTV reduction were 28%, 40%, and 65%, respectively (Figure 2). The geometry of the sCT in each treatment session resembled the simulation CT instead of the on-couch CBCT throughout the treatment course. The side-by-side axial view comparison of the simulation CT, CBCT, and sCT in the 20th treatment session is shown in Figure 3. The overridden volumes in the sCT increased interfractionally, representing growing differences between the sCT and CBCT in each fraction (Figure 1).

Dose difference between the recalculated and original online plans

**Figure 4:**
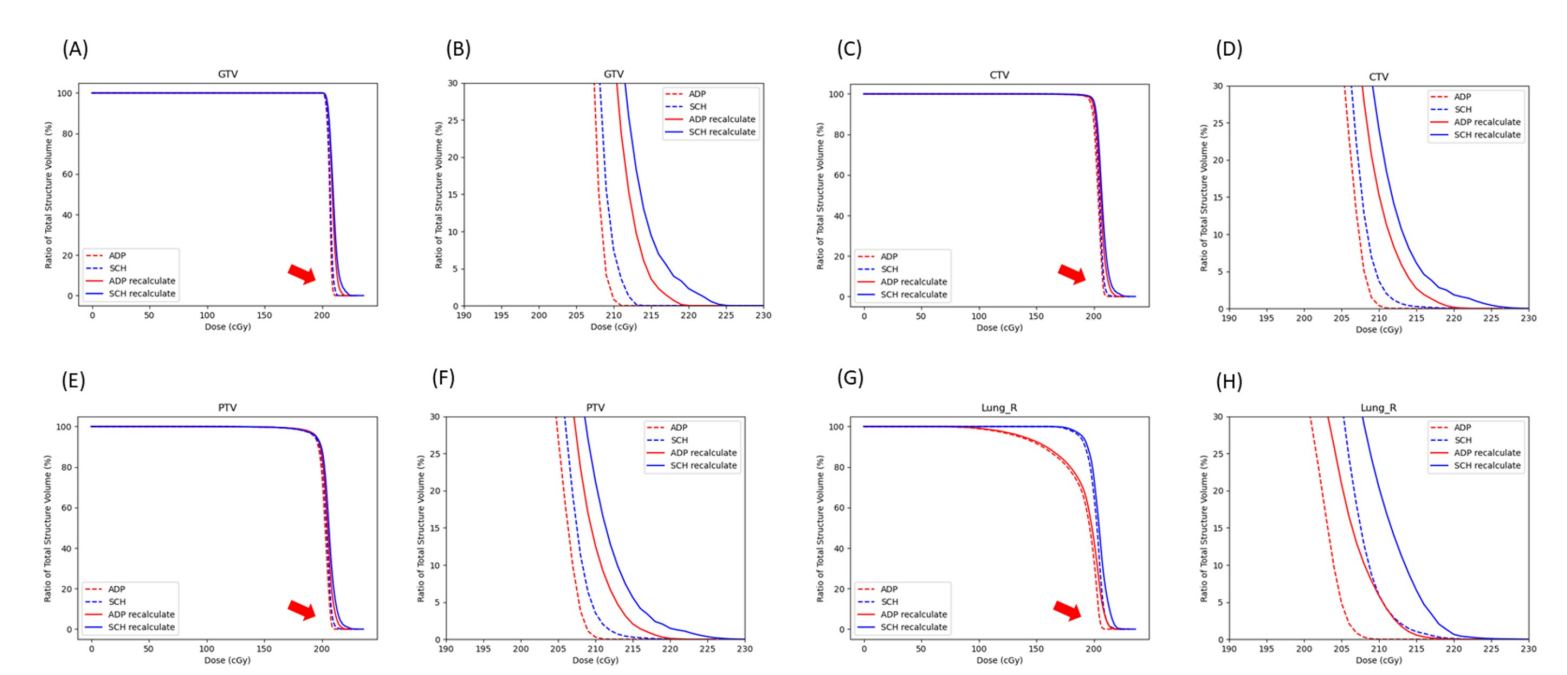
The DVH of the online and recalculated adaptive and scheduled plans. The DVH curves derived from the online adaptive (red dashed line), online scheduled (blue dashed line), recalculated adaptive (red solid line), and recalculated scheduled (blue solid line) plans for the (A,B) GTV, (C,D) CTV, (E,F) PTV, and (G,H) right lung. (B,D,F,H) are enlarged in the high-dose areas for better visualization. DVH: dose-volume histogram; ADP: adaptive; SCH: scheduled; GTV: gross tumor volume; CTV: clinical target volume; PTV: planning target volume; Lung_R: right lung.

Without overriding the dissipated tumor, the dose received by the targets and right lung was underestimated. All DVH curves shifted to the right after overriding the shrunken tumor on sCT (Figure 4). The difference between the original and recalculated plans was most significant at the high-dose area for the target volumes and right lung.

**Figure 5:**
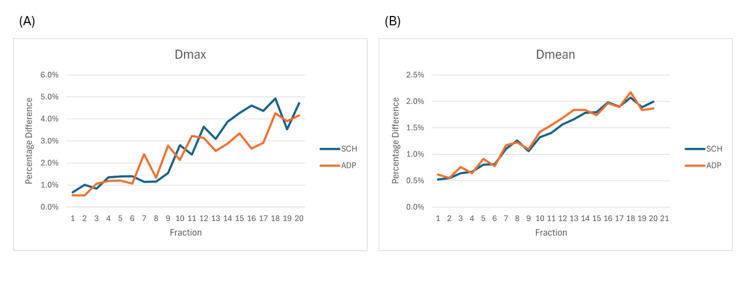
Percentage difference between the online and recalculated plans of the 20 adaptive and 20 scheduled plans. (A) Differences in maximum dose (*D*_max_) between online plans and recalculated plans, presented as mean values presented as mean values of the 20 sessions of SCH and ADP plans. (B) Differences in mean dose (*D*_mean_) between online plans and recalculated plans, presented as mean values of the 20 sessions of SCH and ADP plans. ADP: adaptive; SCH: scheduled.

For the GTV, CTV, PTV, and right lung, recalculated plans had higher *D*_max_ and *D*_mean_ than the original online plans (Table 2). The paired t-test showed a statistically significant underestimation of the dose distribution in the original online plans compared to the recalculated plans. The dose difference between the online and recalculated plans became more prominent fraction after fraction. Initially, the difference in *D*_max_ was approximately 1%, increasing to 5% during the latter half of the treatment course (Figure 5A). Similarly, the difference in *D*_mean_ was approximately 0.5% initially and increased to 2.5% as treatment progressed, with larger discrepancies observed in the latter stages of treatment (Figure 5B).

**Table 2:** The dose difference between the target volumes and the right lung for the online adaptive, online schedule, recalculated adaptive, and recalculated schedule plans. The dose of the twenty sessions is shown as mean ± standard deviation (cGy). Data were analyzed using paired t-tests. SCH: scheduled; ADP: adaptive; GTV: gross tumor volume; CTV: clinical target volume; PTV: planning target volume.

Structure		SCH, online (n=20)	ADP, online (n=20)	SCH, recalculate (n=20)	ADP, recalculate (n=20)
GTV	*D*_mean_	207.1 ± 1.0	206.4 ± 0.8	210.0 ± 2.4	209.1 ± 2.0
	*D*_max_	213.3 ± 0.9	211.4 ± 1.0	220.8 ± 4.2	216.9 ± 2.7
CTV	*D*_mean_	204.5 ± 0.7	203.2 ± 0.2	207.2 ± 1.9	205.8 ± 1.3
	*D*_max_	220.8 ± 4.1	215.6 ± 1.7	226.3 ± 7.2	220.0 ± 3.2
PTV	*D*_mean_	202.9 ± 0.7	201.8 ± 0.1	205.5 ± 1.6	204.2 ± 1.1
	*D*_max_	220.9 ± 4.0	216.4 ± 2.5	226.3 ± 7.2	220.1 ± 3.2
Right lung	*D*_mean_	201.3 ± 2.3	187.3 ± 7.0	203.9 ± 2.9	190.2 ± 6.6
	*D*_max_	220.6 ± 4.0	211.6 ± 0.97	225.42 ± 7.3	218.0 ± 3.9

**Figure 6:**
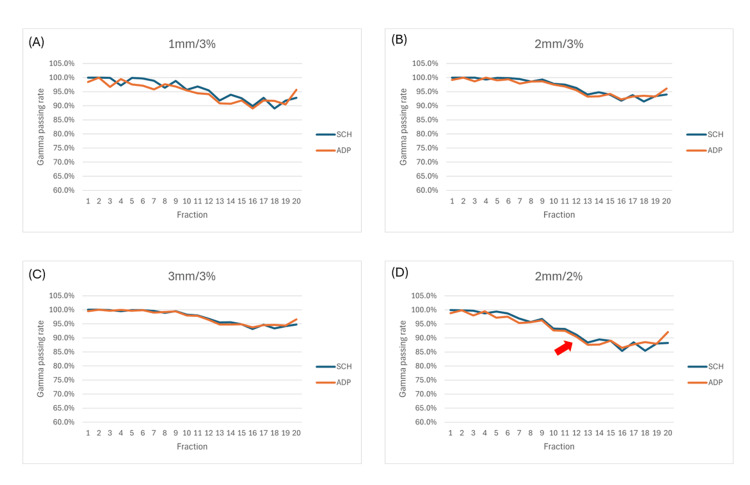
Gamma passing rate of adaptive and scheduled plans using (A) 1 mm/3% criteria, (B) 2 mm/3% criteria, (C) 3 mm/3% criteria, and (D) 2 mm/2% criteria. ADP: adaptive; SCH: scheduled.

The location of the most prominent dose difference between the online and recalculated plans was in the mid-mediastinum, which lies near a significantly shrunken tumor. The distribution of the dose difference and 3D gamma passing rate was also in the same direction as the beam arrangement. Gamma index analysis revealed that in both adaptive and scheduled plans, the passing rates for the criteria of 1 mm/3% (Figure 6A), 2 mm/3% (Figure 6B), and 3 mm/3% (Figure 6C) were consistently above 90%, meeting general clinical standards [10]. However, the passing rate for the 2 mm/2% criterion dropped below 90% after the 12th session (Figure 6D). Additionally, it was observed that after the 10th treatment session, the gamma analysis showed a significant decline under all conditions. When observed alongside volume changes, a reduction of more than 30% in volume was associated with a decrease in gamma passing rates.

## Discussion

In our study, we observed significant inter-fractional tumor volume reduction, with the right lung volume expanding daily. The adaptive plan was selected for every RT session due to its superior dose conformity and lower dose to the spinal cord. However, we encountered a few challenges as the volumes of the tumor and the right lung changed. First, upon examining the sCT after each treatment session, we found that its geometry deviated from the CBCT over time. Second, as time progressed, our confidence in the accuracy of the online adaptive plan evaluation decreased because the sCT more closely resembled the initial simulation CT rather than the daily CBCT. Third, determining the optimal timing for re-simulation was challenging, as the target volume reduced daily compared to the previous treatment session (Figure 1).

To address these challenges, we retrospectively evaluated the treatment plan for each fraction. We overrode the sCT density of the dissipated tumor, recalculated each plan, and plotted the mean and maximal DVH for the target volumes and the right lung for the original online and recalculated adaptive and scheduled plans, which demonstrated a consistent trend. The recalculated plans showed slight underestimations in the dose distribution for the target and the OAR (the right lung). Careful examination of the DVHs revealed that all four graphs in Figure 4 underestimated both *D*_mean_ and *D*_max_, with the latter being underestimated to a greater extent (Table 2; Figure 5). On the other hand, the anatomical location of the most significantly underestimated dose was near the location of the most significant inter-fractional tumor volume dissipation and was on the path of the beam arrangement. This is understandable because the dose underestimation resulted from a change in dose attenuation as the tumor volume shrank. As the tumor dissipated, the attenuation on the beam path decreased, leading to an increased dose distribution in the tissues along the beam path. Additionally, we noted that in this case, when the right lung volume changed by more than 30%, the passing rate dropped below 95% (Figure 6). Although the gamma passing rates for both scheduled and adaptive plans met AAPM standards throughout the treatment process, the passing rates declined with changes in target and/or OAR volumes. Therefore, re-simulation should be considered when there are significant changes in target and/or OAR volumes.

Based on the above findings, for clinical cases receiving oART with significant inter-fractional tumor volume reduction, we concluded that: (1) Both *D*_mean_ and *D*_max_ could be underestimated in the on-couch adaptive plans, with the DVH curve left-shifted compared to the actual delivered dose. (2) The location of hotspots and the volume of the hot area shown on-couch during adaptive plan evaluation may be displaced compared to the actual delivered dose. (3) Significant changes in target and/or OAR volumes may warrant re-simulation to maintain gamma passing rates above 95%.

As tools for personalized radiotherapy have developed in recent years, the use of oART is becoming increasingly common in clinical settings. While new technologies can introduce unexpected challenges, this study emphasizes the importance of carefully evaluating the radiation dose generated based on sCT. Both CBCT-based and MRI-based oART rely on sCT for dose calculation, and its suitability has been a topic of debate since oART was clinically applied [11-14]. This debate arises because sCT is generated through deformation algorithms, which may not always accurately reflect the real anatomical structure that CBCT presents. A recent article [9] on commissioning and dose uncertainty simulated common clinical scenarios such as weight changes, target inter-fraction displacement, and gas changes. In scenarios involving gas changes, it was observed that sCT may not accurately reflect the presence of gas if it was absent during the initial CT simulation. The differences in calculated and measured point doses for gas change scenarios were around 2% to 4% for both scheduled and adaptive treatment plans. In our clinical scenario of tumor shrinkage, the tumor tissue density present during CT simulation dissipated over the course of treatment. In this context, we observed dose differences in treatment plans with and without density overrides. Unlike gas changes that can be overridden with tissue density during treatment planning, tumor volume changes cannot be predicted and thus cannot be overridden at the time of planning. Therefore, real-world clinical cases are essential for understanding the impact of target volume changes on dose distribution.

The lung is a specific tumor site that often necessitates re-simulation and adaptation. Common indications for ART include pleural effusion, atelectasis, tumor regression, and displacement [15,16]. One study reported an average of 38% GTV shrinkage in NSCLC patients receiving definitive RT [16]. While previous studies have evaluated the accuracy of auto-contouring and resulting dosimetry in lung oART, they have not thoroughly examined the accuracy of sCT generation or its dosimetric impact [8]. Another study assessed the suitability of deformable image registration (DIR) software by comparing the dose of planning CT and sCT for locally advanced head and neck cancer [13]. It was found that when patient contour changes exceeded 1 cm, the DIR software struggled to deform the soft tissue accurately. Other studies have reported similar challenges with DIR image quality when large volume changes occur [17-20].

Our study echoes and complements previous research [13,17-20], providing clinically significant insights for oART users, especially for physicians and physicists treating the lungs. This dosimetric study serves as a valuable reference and guide for oART planning in similar areas, such as pleural metastasis and mesothelioma, and for cases with significant inter-fractional tumor volume shrinkage. The limitations of DIR-generated sCT should be considered during oART treatment delivery when large changes in tumor volume are expected.

## Conclusions

By recalculating the treatment dose after overriding the reduced tumor volume, we concluded that for this clinical case receiving oART with significant inter-fractional tumor volume reduction, both *D*_mean_ and *D*_max_ were underestimated in on-couch adaptive plans, resulting in a left-shifted DVH curve. Additionally, the location and volume of hotspots displayed during on-couch adaptive plan evaluation were displaced compared to the presumably actual delivered dose. Consequently, re-simulation should be considered to maintain gamma passing rates above 95%.

Despite minor discrepancies, the adaptive plans provided a more tailored approach to each session, reducing the irradiated volume of the right lung and maintaining ideal dose coverage of the target volume, all while achieving acceptable passing rates. This highlights the potential of adaptive RT to enhance treatment precision and efficacy in a dynamically changing tumor environment.
